# Lean Management Improves the Process Efficiency of Controlled Ovarian Stimulation Monitoring in IVF Treatment

**DOI:** 10.1155/2022/6229181

**Published:** 2022-03-16

**Authors:** R. Muharam, F. Firman

**Affiliations:** ^1^Division of Reproductive Endocrinology and Infertility, Department of Obstetrics and Gynecology, Faculty of Medicine, University of Indonesia, Jakarta, Indonesia; ^2^Yasmin Infertility Clinic, Dr Cipto Mangunkusumo General Hospital, Jakarta, Indonesia; ^3^Hospital Management Division, Center for Health Service Policy and Management, Faculty of Medicine, Public Health and Nursing, Gadjah Mada University, Yogyakarta, Indonesia

## Abstract

**Background:**

Evaluation of patients' experiences and satisfaction is vital for assessing the quality of healthcare service, including in fertility clinics. One promising concept that has recently been widely used to increase efficiency and service quality in hospitals is the lean concept. Lean is a form of philosophy that focuses on reducing waste of a process and continuous improvement so that consumers receive greater value. This study aims to identify waste and improve efficiency using lean management methods in the controlled ovarian stimulation (COS) monitoring process during in vitro fertilization (IVF) treatment in a fertility clinic.

**Methods:**

This study used an action research approach by observing the total service time of monitoring ovarian stimulation in IVF patients (*n* = 40). The identified waste and solutions were then compiled for use in a focus group discussion (FGD). From the FGD, a priority plan was obtained for the implementation of lean management. This study uses the PDCA cycle for improvement.

**Results:**

Three priority solutions were chosen, which are as follows: (1) evaluating ovarian stimulation via USG only; (2) allocating more time during doctor's counselling; and (3) increasing counselling time by nurses in the injection room. The total patient wait time was reduced to 6 hours 32 minutes over the three visits, 13 hours 35 minutes decrease from before the intervention. In addition, the value-added ratio (VAR) was increased from 9% to 22% after the intervention.

**Conclusion:**

This research provides theoretical and practical contributions for the lean management principles in IVF treatment. The findings of this study will contribute to the pursuit of knowledge and dissemination of lean principles in the management of healthcare, including IVF clinics.

## 1. Introduction

One of the most critical steps in the IVF procedure is the controlled ovarian stimulation (COS) process and its monitoring. Monitoring of COS aims to assess the ovarian response to gonadotropin hormone administration, determine the optimal dose and time of gonadotropin administration, and prevent ovarian hyperstimulation syndrome (OHSS) [1]. Patients undergoing in vitro fertilization (IVF) procedures face both financial and emotional challenges [2]. A high level of stress can negatively affect the outcome of IVF [3]. Poor outcomes caused low satisfaction and reflected poor quality of care. Evaluation of patients' experiences and satisfaction is vital for assessing the quality of healthcare service, including in fertility clinics. Research showed that waiting times, information provision, and emotional support were the least positive aspects of care in fertility clinics and needed to be improved [4].

To maintain the quality of healthcare services, healthcare facilities worldwide are searching for solutions, methods, and strategies that focus on not only reducing costs and waste but also improving patients' safety and satisfaction [5]. One promising concept that has recently been widely used to increase efficiency and service quality in hospitals is the lean concept. Lean is a form of philosophy that focuses on reducing waste of a process and continuous improvement so that consumers receive greater value [6]. Although there are a number of different names for those strategies (lean production, lean/six sigma, Kaizen, etc.). The term “lean” as a concept for management is accepted and widely used. Result from a study conducted in 2007 showed that lean methodologies can indeed be applied and implemented to healthcare. The study reported impressive results, with a 42% reduction in paper work, better multidisciplinary team working, and a reduction in length of stay by 33% [7]. Moreover, some studies also shown that implementing lean methodologies reduce patient waiting times, improve patient flow time, increase efficiency of services, improve patient outcome, and even decrease mortality [8–10]. A recent study also showed that implementation of lean six sigma approach could significantly decrease the risk of healthcare associated infections [11].

This study is a pilot study to test the implementation of lean management at Yasmin Fertility Clinic in Cipto Mangunkusumo General Hospital (RSCM), Jakarta, Indonesia. It offers an example for healthcare managers and executives on how to implement lean management principles, particularly the PDCA cycle approach, to identify and eliminate process waste in their hospitals or clinics. It is a versatile approach and can be implemented in different and unique healthcare settings.

## 2. Materials and Methods

### 2.1. Study Design

This study used an action research approach by observing the total service time of monitoring ovarian stimulation IVF patient services. The observation was carried out from June to September 2019, on 40 patients recruited from the IVF program at Yasmin Fertility Clinic in Cipto Mangunkusumo General Hospital (RSCM), a National Referral Hospital in Jakarta, Indonesia. The observations were conducted on patient service process flow in the IVF clinic and the supporting units involved, and the patient service process flowchart was mapped into spaghetti diagrams and value stream mapping (VSM).

Waste and solutions were identified using the lean management theory. The identified waste and solutions were then compiled for use in a focus group discussion (FGD). From the FGD, a priority plan was obtained for the implementation of lean management of monitoring ovarian stimulation processes. After the implementation phase was complete, reobservations were made to evaluate the results.

In the VSM used in mapping, the service process includes cycle time, value-added (VA) time, and NVA time. Cycle time is the time taken by a clinician to complete providing services to one patient before moving to the next. VA time is the time of the task component recorded on the observation form that results in a change in the patient's condition through diagnosis, pain control, and treatment such that the patient is willing to pay. NVA time is the time component of the task recorded on the observation form that can be identified as waste [12].

### 2.2. Study Sample

The subjects were recruited using purposive sampling. The inclusion criteria for this study were as follows: (1) Yasmin Clinic patients who were in the IVF program from June–September 2019, (2) patients of reproductive age, and (3) patients who gave consent. The exclusion criteria for this study were as follows: (1) partially monitored patients at the Yasmin Clinic, (2) patients who cancelled the IVF cycle, (3) nonresponsive patients, (4) patients who needed a prolonged IVF cycle, and (5) patients with a history of OHSS. A total of 40 participants were recruited, 20 before the intervention (prelean group) and 20 after the intervention (postlean group). The preintervention feedback interview guide is available in the supplementary materials.

### 2.3. Study Variables

The variables that are studied in this research are value added, nonvalue added, value added ratio, waiting time, lead time, and process efficiency in the COS process in Yasmin Clinic. These variables will be evaluated before and after the implementation of lean management. The results of observed variables were represented by creating a value state map for better visualization.

### 2.4. Lean PDCA Cycle

The lean methodological approach that was implemented in this study was the PDCA cycle. The PDCA cycle is an improvement method in lean management. It consists of four steps which are plan, do, check, and action. The “Plan” step is the first step on the PDCA cycle that aims to identify problems, investigate the causes of those problems, and create solutions. After the real problems are revealed, the selected solutions will be tested in the “do” step. The “check” step refers to evaluate the outcomes after the improvement. Finally, the “act” step refers to make adjustments and standardization. Continuous monitoring is also a crucial part of this final step [13].

In this study, the first step was to assess problems and wastes of service in Yasmin Fertility Clinic and discuss the solutions with focused group discussion and scoring method. The selected solutions that are the most suitable and feasible will be implemented for the second step. After the implementation, evaluation will be conducted by reobserving patient services and gathering the postimplementation data. Adjusting of the implementation and continuous monitoring will be conducted as the final step.

### 2.5. Plan Step

#### 2.5.1. Field Observer Recruitment

Two field observers were selected and recruited from a group of nurses who worked in the clinic and understood the service process. They were briefed on how to measure the time of each category (including VA, NVA, and waste) and how to fill in the observation guidelines. Prior to observation, a trial was conducted by assigning a researcher and a field observer to observe the same patient. Both the researcher and the field observer must measure the time of VA, NVA, and waste of three patients. The trial was considered satisfactory when there was no difference, with a tolerance of ±1 second, in filling out the observation instruments and time calculations between the researcher and the field observer.

#### 2.5.2. Waste and Solution Identification Stage

The researchers assigned the data collection tasks to the field observers before each observation time started. Researchers and field observers directly observed the service process of IVF patients by mapping the process flow and recording the time of each subprocess. The first visit is on the second day of menstruation, the second visit on the seventh day of menstruation, and the third visit on the ninth or tenth day of the cycle. The researchers processed the data obtained during the prelean observational stage, including calculating the delay at subprocess points and calculating the waiting time each day. After all the subject data were obtained, the researchers made a current state map of the flow of the IVF patient service processes. Consequently, researchers created a presentation about lean and compiled their proposed solutions to eliminate waste in the process flow.

An FGD was held to identify waste from previously obtained data and to formulate solutions to eliminate waste in the process flow. The team included in the FGD consisted of the heads of RSCM and the Yasmin IVF Clinic unit, the Yasmin IVF clinicians, the nurse coordinator, and administrative staff.

### 2.6. Do Step

#### 2.6.1. Implementation of the Solution

Prioritized solutions are selected with MIV/C scoring. The selected solutions that have the highest score in MIV/C scoring will be implemented in the service processes. Training for the clinic staff will be conducted before the implementation to familiarize them with the selected solutions. The implementation will be conducted for 4 months.

### 2.7. Check Step

#### 2.7.1. Evaluation Stage

After the interventions were implemented and well-run, researchers reobserved the patient service processes during the COS protocol. Researchers analyzed the data obtained using statistical tests; they also conducted in-depth interviews with eight IVF service staff at Yasmin Clinic. Any feedback regarding new process flows and interventions implemented was used to improve the patient service process continuously.

Researchers also assessed the protocol and clinical outcomes of the COS procedure in both pre and postlean groups by evaluating the number of normal oocytes collected, oocyte maturation index, mean duration of stimulation, and mean total dose of gonadotropins administered. The oocyte maturation index is the ratio of inseminated normal metaphase II oocytes to the total number of normal oocytes collected.

### 2.8. Act Step

#### 2.8.1. Adjustment

After the evaluation, adjustments and continuous monitoring will be conducted for the final implementation.

### 2.9. Statistical Analysis

Statistical analysis was conducted with SPSS Statistics software. Descriptive statistical analysis was carried out on the two data groups, the pre and postlean groups, followed by the data normality test. The data normality test was conducted with the Kolmogorov–Smirnov test; the analysis of significance used the Mann–Whitney *test*. A *p*value < 0.05 is statistically significant.

## 3. Results

### 3.1. Characteristics of Research Subjects

The characteristics of the research subjects and their clinical indications for IVF are shown in Table 1, respectively. Among 40 subjects, 18 (45%) were between 30 and 35 years of age, and 17 (42.5%) were primary infertile. The most frequent underlying cause of infertility was sperm abnormality in 16 couples.

### 3.2. Service Process Flow of Patients during COS Protocol

**Figure 1:**
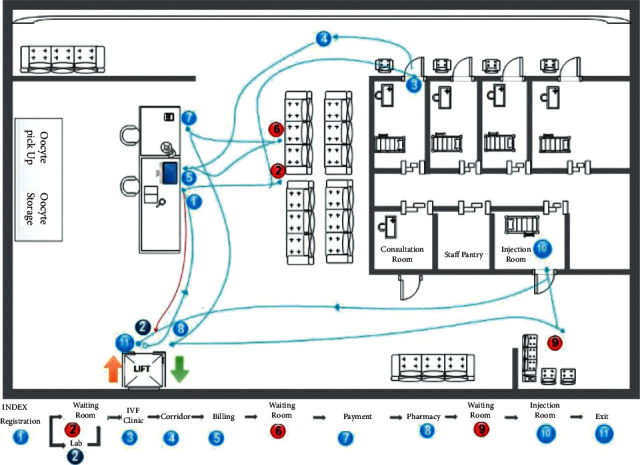
Spaghetti diagram of patient service flow during COS visits in Yasmin Clinic.

Patients underwent the COS protocol, which consists of three visits before oocyte pick-up (OPU). On every visit, it was found that the ovarian stimulation service in Yasmin Clinic before intervention (prelean) consisted of seven main service processes with 18 service subprocesses (Figure 1).

From the observation data, as shown in Table 2, it was found that the longest waiting time was in the subprocess of taking laboratory results, which was 9 hours and 35 minutes per patient, counted from the total of three visits. The longest VA was found in the patient's examination by doctor, in the subprocess, with an average of 30 minutes.

**Figure 2:**
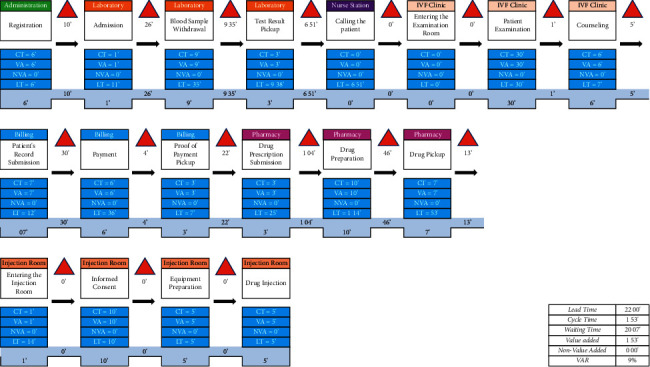
Current state mapping for COS process in Yasmin Clinic.

The result of VSM before intervention (current state mapping) showed the mean waiting time (WA) of patients for all three visits was 20 hours and 7 minutes, with a VA time of 1 hour 53 minutes (Figure 2). This result provides a value-added ratio (VAR) of 9%.

After knowing the baseline data of the service flow at the Yasmin Clinic, an FGD was then conducted to discuss the waste found by the researcher team during the observations (Table 3). The researcher then leads the discussion and asks for opinions and suggestions from all FGD participants to formulate possible solutions.

Determination of priority problems was done using the decision-matrix method. The highest score from importance, technical feasibility, and resource availability (I × *T* × R) will be chosen as the priority problem in the matrix criteria. Three priority issues were selected, namely, the long waiting time for the doctor's arrival, the long waiting time for the laboratory test results, and the nurse's motion waste by providing information to the patient and calling for the next one at the same time (Table 4). Subsequently, researchers analyzed the root causes of these problems with the fishbone diagram.

In accordance to the concept of Kaizen, researchers urge all employees involved in the FGD to provide their views on the problems found and to offer ideas and solutions to solve the problems [14]. The solutions were then prioritized by considering the magnitude of the problem that can be solved (M), the importance of the problem (I), the vulnerability of the solution (V), and the amount of cost required (C). Finally, solutions with the highest MIV/C score were discussed in the FGD with the clinic staff (Table 5) [15].

### 3.3. Lean Management Implementation

**Figure 3:**
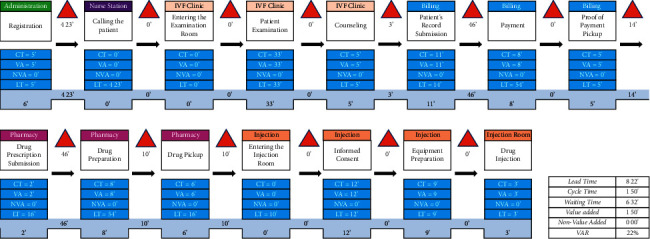
Future stream mapping for COS process in Yasmin Clinic.

To evaluate the success of the interventions, the researchers then conducted another observation, and the results of the time observations were visualized in the form of future state mapping (FSM; Figure 3). From the results of FSM, it was found that the total patient waiting time was 6 hours 32 minutes, 13 hours 35 minutes decrease from before intervention. Total VA after the intervention was 1 hour 50 minutes. There was an increase in VA in the subprocess of informed consent and equipment preparation in the injection room (Table 6) due to additional patient counselling within these processes. The VA also increased in the subprocess of examination by the doctor to 33 minutes from the previous 30 minutes. The VAR value increased by 22% after the intervention.

From the results of the Mann–Whitney test, it was found that the time length differences were significant (*p* < 0.05) on both WA and VA in the subprocesses (Table 7). Intervention was carried out as follows: the laboratory process, the nurse station process, the patient examination subprocess, and the injection room process.

### 3.4. Patient's Clinical Outcomes

Table 7 represents the results of patients' clinical outcomes. The mean gonadotropin doses in the prelean and postlean groups were 3726 ± 1236 IU and 3366 ± 1244 IU, respectively. The difference was found to be not statistically significant (*p* = 0.29). The mean duration of stimulation was also found to be not different between pre and postlean groups (10.33 vs. 10.06, *p* = 0.47).

Two patients could not be analyzed for the outcomes, one in each group. One patient was due to azoospermia (oocyte not inseminated), and the other was due to empty follicle syndrome. The mean numbers of normal oocytes collected were 5.84 ± 4.5 and 10.42 ± 8.1 in the prelean and postlean groups, respectively. However, the oocyte maturation index was higher in the prelean group than in the postlean group (85.7% vs. 79.5%). A nonparametric statistics analysis on both outcome variables showed no statistically significant difference between the pre and postlean groups (*p* > 0.05).

## 4. Discussion

### 4.1. Statement of Principal Findings

The results of time observations were displayed in VSM, which is useful for visually depicting all activities in a process flow [16]. It can also help identify waste and become an effective tool to describe a change in lean management [17]. The VSM before intervention (current state mapping) showed the mean waiting time of patients for all three visits was 20 hours and 7 minutes, with a VA time of 1 hour 53 minutes. This result provides a 9% VAR. From the VSM, it was noted that WA was the longest in the subprocess of obtaining laboratory results, which was 9 hours and 35 minutes.

From the results of future state mapping, it was found that the total patient waiting time was 6 hours and 32 minutes, 13 hours and 35 minutes decrease from before the intervention. The total VA after the intervention was 1 hour 50 minutes, and the VAR value increased to 22%.

### 4.2. Interpretation within the Context of the Wider Literature

From these results, it can be concluded that patients' waiting time was still very long compared to the VA that patients receive. This is still far from the hospital's minimum service standard, which recommends a WA for outpatient services of 60 minutes [18]. Research conducted at the outpatient clinic of Thong Nhat Hospital also showed patient waiting times of around 104.1 minutes [19]. These studies indicate that long waiting times are still a problem in health services, especially in developing countries.

After the implementation of lean management, there was an increase in the VAR value. Service systems that have a VAR of up to 20% have met world standards. An increase in the VAR value proves an improvement in service efficiency [20].

Our findings are consistent with other studies. A study conducted at an outpatient clinic of a teaching hospital in Brazil showed that implementing lean theories could reduce patient waiting times by approximately 4 hours. By reducing patient waiting time, patients' and health workers' satisfaction increased [21]. Research conducted at tertiary hospitals in Abu Dhabi also showed a decrease in waiting time from 40–60 minutes to 4–6 minutes after using lean management [22]. A study conducted at a reproductive clinic in China also found that the application of PDCA cycle reduced patient waiting time in outpatient procedures by 128.83 minutes and proved to be statistically significant. The study also found an increase in patients' satisfaction by 24.07 points after the application [23].

A meta-analysis study compared hormonal examinations and ultrasound examinations for ovarian monitoring. This study stated that antral follicular counts using ultrasound was the best predictor of ovarian stimulation outcome in IVF because it predicted a poor response to ovarian stimulation better than hormonal [24]. The estimation of ovarian response using ultrasound is easier and more reliable because, in addition to seeing the quantity of follicles, ultrasound can also see the quality of the follicles. The use of ultrasound in IVF further reduces costs, saves time, and improves accessibility [25]. In our clinic, the elimination of hormonal laboratory tests can save up to IDR 2,342,000 (∼ USD 163) of the patient's money. From these studies, it can be concluded that the elimination of laboratory tests in the ovarian stimulation process is feasible.

This study found that the waiting time decreased from 20 hours to 7 minutes to 6 hours and 32 minutes. Shortening the patient's waiting time can help reduce the patient's psychological stress and anxiety [26]. A review of lean also states that implementing lean in health services increases patient satisfaction and reduces patient stress [27]. This is expected to increase the IVF success rate.

In the fertility care aspect, specifically IVF, this study showed that eliminating laboratory tests in the ovarian stimulation process is feasible. It does not negatively affect the COS procedure quality and safety. It also reduces costs and saves time for both the health workers and the patients.

## 5. Conclusion

Using action research study design, we gained in-depth knowledge of the problem in our clinic by obtaining both qualitative and quantitative data. Therefore, the intervention can be tailored to our specific problems and resources. We also laid out a detailed step-by-step of the PDCA cycle approach. We believe this method can be replicated and implemented by other researchers or managers of healthcare facilities.

An improvement of service proven by an increase of VAR in this study suggests that lean management is recommended for health services, including fertility clinics, because it is easy and affordable to implement. In addition, by using lean management, clinical procedures can be conducted more efficiently.

This study has several limitations. Due to limited time and the number of patients in our clinic, we could not select patients based on their medical condition rigorously.

The limited time allocated for the study also influences the choices of intervention. We prioritized interventions in which changes could be made quickly. Due to this reason, we also did not analyze the long-term satisfaction of patients and health workers involved in the lean management implementation.

## Figures and Tables

**Table 1:** Characteristics of subjects.

Characteristics		Prelean	Postlean
Age	<30	0 (0%)	2 (5%)
	30–35	10 (25%)	8 (20%)
	36–40	8 (20%)	6 (15%)
	>40	2 (5%)	4 (10%)

Fertility	Primary infertility	17 (42.5%)	17 (42.5%)
	Secondary infertility	3 (7.5%)	3 (7.5%)

Total rFSH (IU)a	Mean^*∗*^	2613 ± 536	2573 ± 760
	Max value	3900	4800
	Min value	1800	1575

Follicle diameter (mm)	Mean^*∗*^	20 ± 2	20 ± 2
	Max value	24	22
	Min value	16	16

Clinical indication^*∗∗*^	Polycystic ovarian syndrome	1	2
	Unexplained infertility	2	3
	Ovarian cyst	6	1
	Sperm abnormality	7	9
	Adenomyosis	1	1
	Endometriosis	5	0
	Diminished ovarian reserve	0	2
	Tubal factor	1	1
	Poor ovarian responder	2	5
	Uterine myoma	0	1
	Serodiscordant couple	1	1
	Antisperm antibodies+	1	1
	Sex selection	1	0

^a^rFSH IU: recombinant follicle-stimulating hormone/international units. ^*∗*^Data presented as the mean ± SD. ^*∗∗*^Patients can have more than one condition.

**Table 2:** COS process in Yasmin Clinic.

No.	Process	Subprocess	Waiting	CT		Lead time
				VA	NVA	
1.	Administration	Registration	0 : 00	0 : 06	0 : 00	0 : 06

2.	Laboratory	Admission	0 : 10	0 : 02	0 : 00	0 : 12
		Blood sample withdrawal	0 : 26	0 : 09	0 : 00	0 : 35
		Test result pickup	9 : 35	0 : 03	0 : 00	9 : 38
3.	Nurse station	Calling the patients	6 : 51	0 : 00	0 : 00	6 : 51

4.	IVF clinic	Entering the room	0 : 00	0 : 00	0 : 00	0 : 00
		Patient examination	0 : 00	0 : 30	0 : 00	0 : 30
		Counselling	0 : 01	0 : 06	0 : 00	0 : 07

5.	Payment	Patient's record submission	0 : 05	0 : 07	0 : 00	0 : 12
		Payment	0 : 30	0 : 06	0 : 00	0 : 36
		Proof of payment pickup	0 : 04	0 : 03	0 : 00	0 : 07

6.	Pharmacy	Drug prescription submission	0 : 22	0 : 03	0 : 00	0 : 24
		Drug preparation	1 : 04	0 : 10	0 : 00	1 : 14
		Drug pickup	0 : 46	0 : 07	0 : 00	0 : 53

7.	Injection room	Entering the room	0 : 13	0 : 01	0 : 00	0 : 14
		Informed consent	0 : 00	0 : 10	0 : 00	0 : 10
		Equipment preparation	0 : 00	0 : 05	0 : 00	0 : 05
		Drug injection	0 : 00	0 : 05	0 : 00	0 : 05
TOTAL			**20:07**	**01:53**	**0:00**	**22:00**
VAR score			9%			

^*∗*^CT: cycle time; IVF: in vitro fertilization; NVA: nonvalue added; VA: value-added; VAR: value-added ratio. Bold text represents total value of waiting time, VA, NVA, and lead time.

**Table 3:** Results of waste identification in COS service processes in Yasmin Clinic.

No.	Waste category	Problem
1.	Defect	(i) Drug supply sometimes runs out; therefore, patients should search for drugs outside the hospital (ii) Incomplete and unreadable medical records (iii) Simultaneous data input into multiple tabs using the available online systems is not possible (iv) The tubing system for speedy delivery of files from laboratories and pharmacies was broken
2.	Overproduction	(i) Inefficient patient registration flow because IVF patients have to register on the ground floor and confirm registration to the administration on the fourth floor of the Yasmin Clinic (ii) Patients were called repeatedly because they were waiting in different places (iii) Rewrite results that are already in the medical record
3.	Waiting	(i) Patients must wait for doctor's arrival (ii) Waiting for medical intervention (drug injection or blood sampling) (iii) Waiting for additional examination results (laboratory result) (iv) Waiting for data input into the online system (v) Waiting for prescription input into the online system (vi) Patients must wait for drugs preparation because they can coincide with patients from other clinics in RSCM
4.	Nonutilized personnel	(i) Limited number of nurses take blood samples, causing long queues (ii) Limited number of nurses in the injection room
5.	Transportation	(-)
6.	Inventory	(-)
7.	Motion	(i) Administration office and examination room are located on different floors (ii) Laboratory and the consultation room are located on different floors (iii) The nurses go back and forth to provide information to the previous patient and call the next patient at the same time (iv) The pharmacy and the consultation room are located on different floors
8.	Extra processing	Estradiol laboratory test on every visit

IVF: in vitro fertilization; RSCM: Cipto Mangunkusumo General Hospital; (-): no problem.

**Table 4:** Solutions based on results from the focus group discussion.

Problem	Solution
1. Waiting for the doctor's arrival for an extended time	(i) Increase patients' value-added by adding time to the doctor's examination (ii) Doctor is asked to arrive on time (iii) Discuss and schedule fixed hours of practice with doctors working in Yasmin Clinic (iv) Recruit more doctors as substitute doctors to take over the morning shift

2. Waiting for the results of the diagnostic workups for an extended time	(i) Ovarian stimulation method can be satisfactorily evaluated by USG; thus, laboratory tests can be eliminated (ii) Collaboration with laboratories outside the hospital for blood tests (iii) Advance notice when a reagent in the laboratory is out of stock

3. The nurse's wasted motion back and forth to provide information to the patient and calling new patients at the same time	(i) Adding a loudspeaker or patient queue number screen outside the examination room; thus, the patient can be called from inside the room (ii) Counselling by a nurse is done in a separate room (iii) Assign a separate nurse to provide counselling for patients to increase value added (iv) Counselling can be continued in the injection room (v) Counselling can be done when patients wait for diagnostic workups

**Table 5:** Prioritized solutions based on MIV/C scoring.

No	Solution	M	I	V	C	Total (MIV/C)
Problem 1: waiting for the doctor's arrival for an extended time						
**1**	**Increase patients' value-added by adding time to doctor's examination** ^*∗*^	**3**	**3**	**3**	**2**	**13.5**
2	Doctor is asked to arrive on time	1	3	2	2	3
3	Discuss and schedule fixed hours of practice with doctors working in Yasmin Clinic	2	2	2	1	8
4	Recruit more doctors as substitute doctors to take over the morning shift	4	2	2	3	4.3
Problem 2: waiting for the results of the diagnostic workups for extended time						
**1**	**Ovarian stimulation method can be satisfactorily evaluated by USG; thus, laboratory tests can be eliminated** ^*∗*^	**3**	**4**	**2**	**2**	**12**
2	Collaboration with laboratories outside the hospital for blood tests	3	2	2	4	3
3	Advance notice when a reagent in laboratory is out of stock	3	2	2	2	6
Problem 3: the nurse's wasted motion back and forth to provide information to the patient and calling new patients at the same time						
1	Adding a loudspeaker or patient queue number screen outside the examination room; thus, patients can be called from inside the room	4	3	2	3	8
2	Counselling by a nurse is done in a separate room	4	2	2	3	5,3
3	Assign separate nurse to provide counselling for patients to increase value-added	4	3	2	2	12
**4**	**Counselling can be continued in the injection room** ^*∗*^	**4**	**3**	**3**	**2**	**18**
5	Counselling can be done when patients wait for diagnostic workups	3	2	2	1	12

^*∗*^Bold text showed solutions with highest MIV/C score for each problem. M: magnitude; I: importance; V: vulnerability; C: cost.

**Table 6:** Results of observation of time before and after lean management.

No	Process	Subprocess	Before lean management				After lean management			
			Waiting	CT		Lead time	Waiting	CT		Lead time
				VA	NVA			VA	NVA	
1.	Administration	Registration	0 : 00	0 : 06	0 : 00	0 : 06	0 : 00	0 : 05	0 : 00	0 : 05
2.	Laboratory	Admission^*∗*^	0 : 10	0 : 02	0 : 00	0 : 12	—	—	—	—
		Blood sample withdrawal^*∗*^	0 : 26	0 : 09	0 : 00	0 : 35	—	—	—	—
		Test result pickup^*∗*^	9 : 35	0 : 03	0 : 00	9 : 38	—	—	—	—
3.	Nurse station	Calling the patients	6 : 51	0 : 00	0 : 00	6 : 51	4 : 23	0 : 00	0 : 00	4 : 23
4.	IVF clinic	Entering the room	0 : 00	0 : 00	0 : 00	0 : 00	0 : 00	0 : 00	0 : 00	0 : 00
		Patient examination^*∗*^	0 : 00	**0:30**	0 : 00	0 : 30	0 : 00	**0:33**	0 : 00	0 : 33
		Counselling	0 : 01	0 : 06	0 : 00	0 : 07	0 : 00	0 : 05	0 : 00	0 : 05
5.	Payment	Patient's record submission	0 : 05	0 : 07	0 : 00	0 : 12	0 : 03	0 : 11	0 : 00	0 : 14
		Payment	0 : 30	0 : 06	0 : 00	0 : 36	0 : 46	0 : 08	0 : 00	0 : 54
		Proof of payment pickup	0 : 04	0 : 03	0 : 00	0 : 07	0 : 00	0 : 05	0 : 00	0 : 05
6.	Pharmacy	Drug prescription submission	0 : 22	0 : 03	0 : 00	0 : 24	0 : 14	0 : 02	0 : 00	0 : 16
		Drug preparation	1 : 04	0 : 10	0 : 00	1 : 14	0 : 46	0 : 11	0 : 00	0 : 57
		Drug pickup	0 : 46	0 : 07	0 : 00	0 : 53	0 : 10	0 : 06	0 : 00	0 : 16
7.	Injection room	Entering the room	0 : 13	0 : 01	0 : 00	0 : 14	0 : 10	0 : 00	0 : 00	0 : 10
		Informed consent	0 : 00	0 : 10	0 : 00	0 : 10	0 : 00	0 : 12	0 : 00	0 : 12
		Equipment preparation^*∗*^	0 : 00	**0:05**	0 : 00	0 : 05	0 : 00	**0:09**	0 : 00	0 : 09
		Drug injection	0 : 00	0 : 05	0 : 00	0 : 05	0 : 00	0 : 03	0 : 00	0 : 03
Total			20 : 07	01 : 53	0 : 00	22 : 00	6 : 32	1 : 50	0 : 00	8 : 22
**VAR score**			9%				22%			

^*∗*^Bold text showed the subprocesses which lean management implemented. CT: cycle time; IVF: in vitro fertilization; NVA: nonvalue added; VA: value-added; VAR: value-added ratio.

**Table 7:** Results of patients' clinical outcomes.

Outcomes	Prelean	Postlean	*p*value
Gonadotropin doses	3726 ± 1236	3366 ± 1244 IU	0.29
Duration of stimulation	10.33	10.06	0.47
Numbers of oocytes	5.84 ± 4.5	10.42 ± 8.1	>0.05
Oocytes maturation index	85.7%	79.5%	>0.05

## Data Availability

The original data generated in this study are available from the corresponding author upon request.

## Supplementary Materials

Supplementary Materials File S_1 .doc is about preintervention feedback interview guide in Bahasa. File S_2 .doc is about preintervention feedback interview guide in English.
