# Supplementary material for: Lean Management Improves the Process Efficiency of Controlled Ovarian Stimulation Monitoring in IVF Treatment
Source: J Healthc Eng. 2022 Mar 16;2022:6229181. doi: 10.1155/2022/6229181 (PMC8942643; doi:10.1155/2022/6229181)
Supplement: Supplementary Materials — File S_1 .doc is about preintervention feedback interview guide in Bahasa. File S_2 .doc is about preintervention feedback interview guide in English. [file 6229181.f1.zip › 6229181.f1/Supplement_1.docx]

Panduan Wawancara Umpan Balik Pra Intervensi

Hari, tanggal :

Waktu :

Tempat :

Jabatan :

Pertanyaan :

1. Bagaimana pendapat Anda terkait kecepatan pelayanan terhadap pasien FIV saat ini?
2. Apakah yang menjadi kendala dalam pelaksanaan proses pelayanan pasien FIV? Mengapa hal tersebut menjadi kendala?
3. Apakah proses pada proses pelayanan pasien FIV masih perlu dilakukan perbaikan? Mengapa? Apa usulan Anda?
4. Apakah proses pelayanan pasien FIV memenuhi harapan Anda? Mengapa?

Panduan Wawancara Umpan Balik Pasca Intervensi

Hari, tanggal :

Waktu :

Tempat :

Jabatan :

Pertanyaan :

1. Bagaimana pendapat Anda terkait kecepatan pelayanan terhadap pasien FIV sebelum dan sesudah perubahan?
2. Bagaimana pendapat Anda tentang perubahan / perbaikan pada proses pelayanan pasien FIV?
3. Apakah yang menjadi kendala dalam pelaksanaan perubahan pada proses pelayanan pasien FIV ? Mengapa hal tersebut menjadi kendala?
4. Apakah proses pada proses pelayanan pasien FIV yang sudah mengalami perubahan ini masih perlu dilakukan perbaikan? Mengapa? Apa usulan Anda?
5. Apakah proses pelayanan pasien FIV setelah intervensi memenuhi harapan Anda? Mengapa?
