# Supplementary material for: Lean Management Improves the Process Efficiency of Controlled Ovarian Stimulation Monitoring in IVF Treatment
Source: J Healthc Eng. 2022 Mar 16;2022:6229181. doi: 10.1155/2022/6229181 (PMC8942643; doi:10.1155/2022/6229181)
Supplement: Supplementary Materials — File S_1 .doc is about preintervention feedback interview guide in Bahasa. File S_2 .doc is about preintervention feedback interview guide in English. [file 6229181.f1.zip › 6229181.f1/Supplement_2.docx]

***Pre-Intervention Feedback Interview Guide***

Date :

Time :

The place :

Position :

Questions :

a. What do you think about the current speed of service to IVF patients?

b. What are the obstacles in the implementation of the IVF patient care process? Why is this a problem?

c. Does the process of providing IVF patient care still need to be improved? Why? What is your suggestion?

d. Did the IVF patient care process meet your expectations? Why?

***Post-Intervention Feedback Interview Guide***

Date :

Time :

The place :

Position :

Questions :

a. What do you think about the speed of service to IVF patients before and after the intervention?

b. What do you think about the changes/improvements to the IVF patient care process?

c. What are the obstacles in implementing changes to the IVF patient care process? Why is this a problem?

d. Does the process in the service for IVF patients that have undergone these changes still need improvement? Why? What is your suggestion?

e. Did the IVF patient care process after the intervention meet your expectations? Why?
